# Draft Genome Sequence of the Panton-Valentine Leucocidin-Producing Staphylococcus aureus Sequence Type 154 Strain NRL 08/001, Isolated from a Fatal Case of Necrotizing Pneumonia

**DOI:** 10.1128/MRA.01299-19

**Published:** 2019-11-21

**Authors:** Adéla Indráková, Ivana Mašlaňová, Ondřej Mrkva, Kamila Bendíčková, Veronika Vrbovská, Jiří Doškař, Roman Pantůček

**Affiliations:** a Department of Experimental Biology, Faculty of Science, Masaryk University, Brno, Czech Republic; University of Rochester School of Medicine and Dentistry

## Abstract

Panton-Valentine leucocidin (PVL)-positive methicillin-resistant Staphylococcus aureus (MRSA) strains cause life-threatening diseases. We present a draft genome sequence of PVL-positive MRSA sequence type 154 (ST154) strain NRL 08/001, isolated from a fatal case of necrotizing pneumonia. The genome consists of 2.9 Mb over 39 contigs and harbors novel composite island staphylococcal cassette chromosome *mec* element (SCC*mec*)-mercury composite type 2B&5.

## ANNOUNCEMENT

Panton-Valentine leucocidin (PVL)-positive strains are often linked with hemorrhagic necrotizing pneumonia (1). Sequencing and genomic analyses of PVL-positive methicillin-resistant Staphylococcus aureus (MRSA) clones will help advance our understanding of the pathogenic potential, evolution, and spread of such superbugs (2).

S. aureus strain NRL 08/001 (=CNCTC 7452), belonging to sequence type 154 (ST154) and *spa* type t667 (3), was isolated from a fatal case of pneumonia in a 22-year-old patient in 2007 in the Czech Republic (4). Only a few PVL-positive ST154 strains have been reported in western Europe (5–7) and Japan (8), but wide distribution of this clone was found in Mongolia (9).

**FIG 1:**
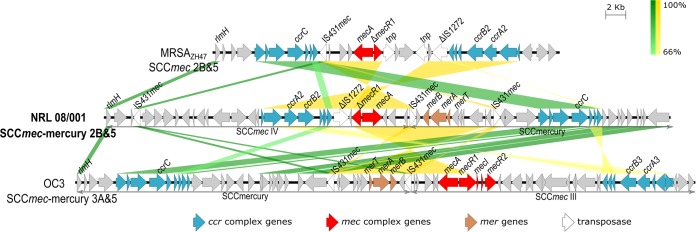
Comparison of the genetic structures of SCC*mec* elements from S. aureus strain MRSA_ZH47_ (ST100, GenBank accession no. AM292304) (18), NRL 08/001, and OC3 (ST239, GenBank accession no. BBKC01000000) (17). Coding sequences are depicted in the direction of transcription as arrows, and *ccr* complex genes (blue), *mec* complex genes (red), a mercury resistance operon (brown), and insertion sequences are annotated. Conserved regions with more than 66% homology are indicated in green, and inversions are indicated in yellow, as determined by BLASTn.

Bacteria were cultured for 18 h at 37°C in 2× YT medium (1.6% [wt/vol] tryptone, 1% [wt/vol] yeast extract, and 0.5% NaCl [pH 7.0]), and genomic DNA was extracted using a High Pure PCR template preparation kit (Roche) with 5 mg/ml lysostaphin added to the suspension buffer. A 400-bp sequencing library was constructed using an Ion Plus fragment library kit (Thermo Fisher Scientific) and sequenced on the Ion Torrent PGM (Thermo Fisher Scientific) using an Ion 318 Chip v2. This generated 5,084,145 single-end reads, for a total of 1.7 Gb, with a mean read length of 335 bp. Default parameters were used for all software, unless otherwise stated. FastQC v0.10.1 was used for read quality assessment. The raw reads were error corrected and assembled using SPAdes v3.13.0 (-k 21,33,55,77,99,127, -iontorrent, -cov-cutoff auto) (10). The final draft assembly contained 44 contigs, of which 39 contigs were longer than 200 bp, covering a total of 2,905,102 bp, with *L*_50_ and *N*_50_ values of 4 and 273,022 bp, respectively, a GC content of 32.73%, and average sequence coverage of 586×. Gene prediction and annotation were performed using RAST v2.0 (genetic code 11, RAST*tk* annotation scheme) (11). The strain contains 2,831 predicted coding sequences and 70 genes for RNAs, of which 61 tRNAs and 1 transfer-messenger RNA (tmRNA) were predicted by ARAGORN v1.2.38 (12), and 5 rRNA operons were estimated from the coverage. Three complete prophages predicted by PHASTER (13) and classified as described previously (14) were found in the genome, a 45.6-kb-long PVL-converting Sa2*int* prophage carrying the PVL locus (*lukS*-PV and *lukF*-PV), a 42.2-kb-long Sa3*int* prophage harboring immune evasion cluster type B (15), and a 42.9-kb-long Sa6*int* prophage disrupted by the insertion of a 13.1-kb transposon upstream of the *terS* gene. The SCC*mec*Finder v1.2 (16) and BLAST-based annotation search identified the unique composite island staphylococcal cassette chromosome *mec* element (SCC*mec*)-mercury type 2B&5 composed of SCC*mec* type IV, an SCC with *ccrC* recombinase, and a mercury resistance operon usually found in SCCmercury adjacent to SCC*mec* III (17) (Fig. 1). Three genomic islands were found in the genome, (i) a 16.3-kb-long S. aureus pathogenicity island (SaPI) carrying genes for enterotoxins C and L inserted into the tmRNA binding protein gene (*smpB*), (ii) a 7.0-kb-long incomplete SaPI with genes encoding a ferrichrome-binding periplasmic protein (*fhuD*) and a potassium uptake protein (*trk*), and (iii) an 18.0-kb-long conjugal transposon, Tn*916,* with the *tetM* resistance gene. Plasmid DNA was found on 5 contigs.

### Data availability.

This whole-genome shotgun project has been deposited in GenBank under accession no. VHNE00000000 (SRA accession no. SRR9600155).
